# Monocyte Activation in Immunopathology: Cellular Test for Development of Diagnostics and Therapy

**DOI:** 10.1155/2016/4789279

**Published:** 2016-01-18

**Authors:** Ekaterina A. Ivanova, Alexander N. Orekhov

**Affiliations:** ^1^Department of Development and Regeneration, KU Leuven, 3000 Leuven, Belgium; ^2^Institute of General Pathology and Pathophysiology, Moscow 125315, Russia; ^3^Institute for Atherosclerosis Research, Skolkovo Innovation Center, Moscow 121609, Russia; ^4^Department of Biophysics, Biological Faculty, Moscow State University, Moscow 119991, Russia

## Abstract

Several highly prevalent human diseases are associated with immunopathology. Alterations in the immune system are found in such life-threatening disorders as cancer and atherosclerosis. Monocyte activation followed by macrophage polarization is an important step in normal immune response to pathogens and other relevant stimuli. Depending on the nature of the activation signal, macrophages can acquire pro- or anti-inflammatory phenotypes that are characterized by the expression of distinct patterns of secreted cytokines and surface antigens. This process is disturbed in immunopathologies resulting in abnormal monocyte activation and/or bias of macrophage polarization towards one or the other phenotype. Such alterations could be used as important diagnostic markers and also as possible targets for the development of immunomodulating therapy. Recently developed cellular tests are designed to analyze the phenotype and activity of living cells circulating in patient's bloodstream. Monocyte/macrophage activation test is a successful example of cellular test relevant for atherosclerosis and oncopathology. This test demonstrated changes in macrophage activation in subclinical atherosclerosis and breast cancer and could also be used for screening a panel of natural agents with immunomodulatory activity. Further development of cellular tests will allow broadening the scope of their clinical implication. Such tests may become useful tools for drug research and therapy optimization.

## 1. Introduction

Immunopathology is associated with the most common life-threatening disorders, including atherosclerosis and related cardiovascular diseases, cancer, and chronic inflammation. A number of diseases, such as lupus erythematosus, rheumatoid arthritis, or HIV infections, are characterized by pronounced immunopathologies; others, such as atherosclerosis and cancer, by less obvious latent pathological changes in the immune system. Such changes may represent early events in the disease initiation and development and might therefore be especially interesting for timely diagnostics and for development of preventive treatment.

The role of the immune system dysfunction in cancer is currently well recognized [1]. Altered macrophage plasticity and polarization can contribute both to the malignancy development and to the tumor vascularization [2]. In that regard, comprehensive analysis of the macrophage population diversity would be necessary for developing adequate therapeutic approaches and monitoring the therapy efficiency.

Recent studies have revealed many aspects of the complex and important role of macrophages in the pathogenesis of atherosclerosis [3]. Formation of the atherosclerotic plaque begins with monocyte activation and transformation into macrophages that reside in the subendothelial area of the blood vessel wall and accumulate lipids in their cytoplasm becoming foam cells. This lipid trapping is performed by means of uncontrolled phagocytosis. At the same time, certain types of macrophages are implicated in tissue repair, and these cells have been found in regressing plaques in mouse models [4, 5]. Therefore, different types of macrophages are responsible for the plaque initiation, growth, and, eventually, regression [6–8]. Correspondingly, anti-inflammatory agents are considered as an important component of antiatherosclerotic therapy [9]. Here again, the analysis of macrophage phenotypic diversity could improve the understanding of the pathological process and assessment of the therapy efficiency.

According to current epidemiological data, atherosclerosis-related diseases and cancer are the two greatest contributors to the overall mortality in the developed countries [10, 11]. Given that these diseases are tightly associated with immunopathology, development of comprehensive diagnostic methods and therapeutic approaches to modulate the immune system appears to be of the greatest importance. However, the existing diagnostic methods are imperfect and their improvement remains challenging. Likewise, no drugs are available to date that allow targeted immune correction in atherosclerosis. It is clear that changes in cytokine expression and phenotypic features of macrophages may reflect the disease progression state. These features may therefore be used for monitoring the pathological process and treatment efficiency.

## 2. Cellular Tests for Diagnostics and Drug Research

In many pathological conditions, the analysis of different types of cells circulating in the bloodstream can provide valuable information about the disease progression. During the recent years, a number of cell types have been isolated and studied for possible application in diagnostics and drug development.

Circulating tumor cells (CTCs) can be extracted from patient's blood and used to analyze the expression of relevant genes and surface markers. For instance, successful isolation and molecular characterization have been described for metastatic breast cancer [12], metastatic colorectal cancer [13], and lung cancer [14]. This strategy is especially useful in cases of advanced metastatic cancer, where the patients could benefit from a personalized treatment. The analysis of CTCs has a great diagnostic potential but can also help in revealing the possible drug resistance of the tumor and designing the optimal therapy [15]. Many current studies are focused on the improvement of CTC-based analyses and their clinical implementation.

Peripheral blood mononuclear cells (PBMCs) are relatively easily obtainable cells that can be used for monitoring a wide spectrum of conditions and pathologies. The analysis of mRNA profiles of isolated PBMCs could be used for evaluation of metabolic changes [16]. PBMCs can also be kept in short-term culture and used for studying cytokine production induced by stimulation. For instance, changes in proinflammatory cytokine production by isolated PBMCs have been described in such conditions as allergies, alterations of immune response, and immunization [17–19]. Studies on PBMCs have demonstrated that vascular endothelial growth factor (VEGF) production was decreased in women with preeclampsia [20]. Isolated PBMCs can serve as a relevant system for testing various drugs, especially related to inflammation [21]. Recently, the potential of macrophage-based test system for diagnostics and treatment of atherosclerosis has been explored [22].

Atherosclerosis is associated with life-threatening cardiovascular diseases [7]. Atherosclerosis progression is usually slow, and the disease often remains asymptomatic until the ischemia of organs and tissues becomes evident. This may happen due to the obstruction of a blood vessel with growing atherosclerotic plaque or the embolism caused by a thrombus formed on a destabilized plaque. Therefore, the first manifestations of the disease are often lethal [23, 24]. Diagnostic of preclinical (asymptomatic) atherosclerosis is therefore especially important. However, it is hindered by the absence of clinical symptoms and complaints and by the fact that the spectrum of risk factors is very wide, including genetic predisposition, lifestyle and diet patterns, chronic inflammation, and metabolic factors. Immunopathology is likely to be one of the mechanisms underlying atherosclerosis development starting from the early stages, and its assessment can therefore have an important diagnostic value.

## 3. Monocyte/Macrophage Diversity and Functions

Monocytes and macrophages are the key players in the innate immune system. These cells can eliminate pathogens by phagocytosis, release of reactive oxygen species, production of proinflammatory cytokines, and modulation of the T-cell immune response [25]. Macrophages are present in all organs and tissues and represent the first line of immune defence, responsible for removal of foreign agents and pathogens. They participate in all stages of the inflammatory process. The pool of macrophages remains constant in every tissue, and the cells are renewed from the population of circulating monocytes [26], although the results of recent studies suggest that these cells are also capable of self-renewal [27]. It has been known for a long time that changes in the phagocytic activity of macrophages might be dependent on changes in the peripheral blood monocyte population, and alterations of the monocyte pool lead to various pathological conditions [28]. Proliferation of promonocytes, which can be stimulated by systemic inflammatory stimuli, leads to the increase of the number of circulating monocytes [29, 30]. Monocytes and macrophages, together with their precursors and dendritic cells, form the mononuclear phagocyte system (MPS) [31], although the identity of the dendritic cells remains disputed [32, 33]. The development, maintenance, differentiation, and function of MPS are regulated mostly by colony-stimulating factor 1 (CSF-1) in homeostatic conditions [34] and by granulocyte-macrophage colony-stimulating factor (GM-CSF) during inflammation [35]. Inflammatory signals and various pathological conditions, including atherosclerosis development, stimulate the inactive circulating monocytes to become activated macrophages that can be distinguished by their phenotypic properties. Macrophages can acquire different functional phenotypes influenced by the surrounding microenvironment in a process known as macrophage polarization.

**Figure 1:**
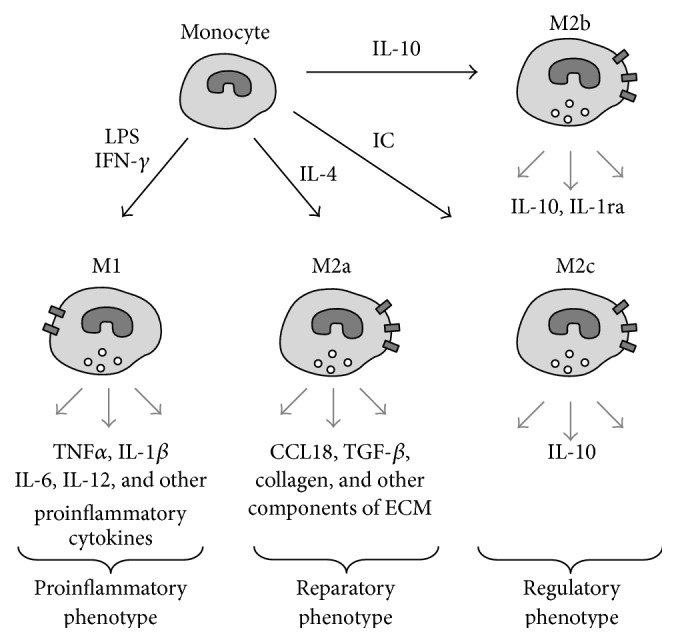
Simplified scheme of macrophage polarization. Activation of monocytes is followed by the polarization of macrophages to acquire proinflammatory phenotype (M1) or anti-inflammatory phenotypes (M2a–M2c) depending on the activation stimuli. Each phenotype is characterized by the secretion of a distinct pattern of pro- or anti-inflammatory cytokines and other molecules. For instance, M1 macrophages release TNF*α*, IL-1*β*, IL-6, IL-12, and other proinflammatory cytokines, whereas M2a macrophages produce CCL18, TGF-*β*, collagen, and other extracellular matrix components. LPS: bacterial lipopolysaccharides; IC: immune complexes; IFN-*γ*: interferon gamma; IL: interleukin; TNF*α*: tumor necrosis factor-alpha; TGF-*β*: transforming growth factor beta; CCL18: CC chemokine ligand 18.

Studies of macrophage population revealed significant heterogeneity and plasticity of this cell type. Reaching consensus on macrophage classification was challenging due to the high variety of activation types, dependence of the results on the particular experimental setup, and differences of macrophage activation profiles between humans and animal models. Recently, a group of leading immunologists have summarized the current knowledge on the issue and drawn recommendations for conducting and reporting the experiments involving macrophage polarization [36]. Initially, two main classes of macrophages, M1 and M2, have been defined which could be obtained by activation of macrophages by proinflammatory interferon *γ* (IFN-*γ*) and lipopolysaccharide (LPS) or by interleukin-4 (IL-4), respectively [37–39]. A simplified scheme of macrophage polarization is presented in Figure 1.

M1 macrophages are characterized by the production of proinflammatory cytokines tumor necrosis factor-*α* (TNF*α*), IL-1*β*, IL-6, IL-12, and proteolytic enzymes, as well as by the expression of Fc-*γ* receptors on the cell surface [40, 41]. Polarization towards the proinflammatory phenotype can be induced* in vitro* by toll-like receptor (TLR) ligands, including TNF*α*, lipopolysaccharide (LPS), and interferon *γ* (IFN-*γ*) that might also play a role in the pathogenesis of atherosclerosis [42]. It has been demonstrated that M1 macrophages are present in the atherosclerotic plaques where they maintain the local inflammatory process and promote the extracellular matrix degradation contributing to the formation of unstable plaques that can induce thrombus formation and are therefore especially dangerous [43–45].

The subpopulation of M2 macrophages was further divided into several subtypes depending on the activation stimuli (Figure 1): M2a (activated by IL-4), M2b (activated by immune complexes), and M2c (activated by IL-10) [46]. Each of these subtypes is characterized by a distinct pattern of cytokine and surface marker expression which can also vary between the species. It has been proposed therefore to refer to different subtypes of macrophages indicating the activation type (e.g., M(IL-4) instead of M2a) [36]. M2a macrophages have strong anti-inflammatory properties and can be regarded as tissue-repairing cells [47]. They have poor phagocytic capacity and participate in the formation of extracellular matrix by stimulating production of collagen. They express IL-1 receptor antagonist (IL-1ra) and secrete CCL18, transforming growth factor *β* (TGF-*β*), and remodelling enzymes. M2b and M2c macrophages express different chemokine receptors, produce IL-10, and can modulate inflammation but do not synthesize the extracellular matrix and can therefore be regarded as regulatory macrophages [48]. Anti-inflammatory macrophages were also shown to express mannose receptor (CD206), stabilin-1, and decoy receptor IL-1RII on the surface [49–51]. M2 phenotype can also be induced by T regulatory cells [52]. However, the heterogeneity of this population requires further studies and standardization of the nomenclature. For the sake of simplicity, later in this work, we will refer to the IL-4-activated M2a macrophages as “M2 phenotype.”

The production and release of the proinflammatory cytokines, such as IL-1*β* and IL-18, are dependent on the macrophage inflammasome status [53, 54]. Inflammasome is a caspase-activating complex formed by several proteins, including caspase-1, which is responsible for cytokine maturation. Active caspase-1 can also be released from the activated cells and may contribute to the damage of neighbouring cells [55]. The inflammasome activation has been reported in various pathological conditions and infections. In can be induced by danger- and pathogen-associated molecular patterns (DAMPs and PAMPs) [56–59]. Importantly, inflammasome is activated in atherosclerosis in response to cholesterol accumulation in the blood vessel wall and formation of cholesterol crystals in foam cells [60]. On the other hand, atherogenesis can be associated with ongoing infections with various pathogens, such as* Chlamydia pneumonia* and* Helicobacter pylori*, which can induce the inflammasome activation [61–63]. There might exist other factors that contribute to the inflammasome activation and atherosclerosis progression, including the formation of uric acid crystals [64] and impaired autophagy [65]. Therefore, the inflammasome activation and release of proinflammatory cytokines and caspase-1 are relevant for atherosclerosis progression and can be regarded as important markers of the pathological process.

## 4. Cellular Tests Based on Monocyte/Macrophage Phenotypic Changes in Atherosclerosis

Development of a reliable monocyte/macrophage-based functional test remains challenging due to several technical problems. Isolation of monocytes from blood can lead to their activation. A number of different isolation methods have been proposed during the recent years. The traditional method implies cell adhesion, which leads to monocytes activation and is therefore widely criticized. Another method is fluorescence-activated cell sorting (FACS), which is fast and accurate but requires labelling of cells with specific antibodies, which can also lead to activation. A pure fraction of monocytes/macrophages can be isolated using magnetic separation. In this method, unspecific activation can also occur due to possible phagocytosis of paramagnetic particles. So far, the only method that can extract nonactivated monocytes is elutriation [66, 67]. It requires, however, special equipment and is impossible to introduce into routine clinical practice. Because of these technical problems, recent studies focused on the identification of molecular markers that could substitute for functional tests in diagnostics of immunopathologies.

Studies of monocyte function include the assessment of their motility, adhesion, phagocytic activity, and low-density protein (LDL) uptake [68, 69]. Macrophages can take part in pathological processes via stimulation with circulating soluble activation factors, adhesion to the endothelium, and migration into the tissue where they meet local activation factors. The monocytes' response to these stimuli depends on their priming in circulation and therefore can have a diagnostic potential. The analysis of macrophage pro- and anti-inflammatory phenotypic classes can provide important information on the disease progression, as has been demonstrated for atherosclerosis [22].

**Figure 2:**
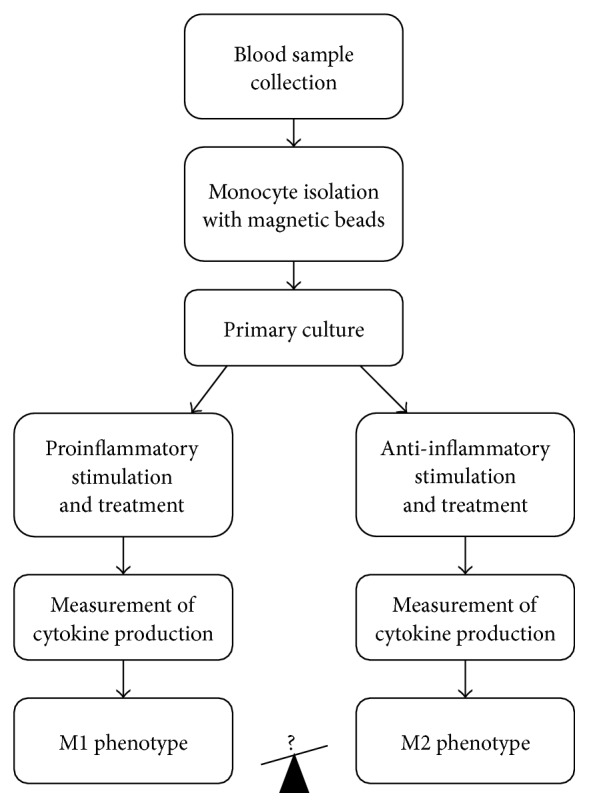
General design of the monocyte/macrophage activation assay.

A monocyte/macrophage-based assay has recently been designed to evaluate changes in monocyte response to pro- and anti-inflammatory stimuli and to reveal possible bias of the macrophage polarization towards M1 or M2 phenotype. In this method, a pure population of blood monocytes was isolated using magnetic separation [49, 70] (Figure 2). The analysis of macrophage activity was performed by stimulating the cells with LPS, proinflammatory stimulus IFN-*γ*, and anti-inflammatory stimulus IL-4 [71]. Pro- and anti-inflammatory cytokine production measurement was used as readout. Proinflammatory activity of macrophages was assessed by the levels of secreted TNF*α* and IL-1*β* and anti-inflammatory activity by the levels of CCL18 production and IL-1ra expression. Inflammasome activation can also be assessed in this experimental system by measuring the IL-1*β* expression at mRNA level and comparing the results with the amount of mature IL-1*β* detected by ELISA. Another evidence of inflammasome activation that can be used as readout is the release of active caspase-1 [55]. Expression and release of the inflammasome-dependent cytokines TNF*α* and IL-8 should also be measured to provide the control of inflammasome activity. Further characterization of macrophages can be performed by analyzing such markers as MMR, CD163, TGF-RII, CSFR1, TNFRI, CD16, CD32, CD64, and stabilin-1, as well as the expression of TLR1, TLR2, and TLR4 at mRNA level and on the cell surface. Changes in the expression of these markers correlate with stimulation type and intensity. The described experimental system can provide important information on monocyte activation state and possible skew of the monocyte predisposition towards pro- or anti-inflammatory response. Apart from altered polarization towards one or the other phenotype, pathological conditions can be associated with other phenotypical alterations of monocytes/macrophage, including phagocytosis, migration, and proliferation. Alterations of macrophage phenotype and plasticity associated with atherosclerosis have recently been discussed in a comprehensive review [46].

The described method has been used to analyze activation of monocytes isolated from blood of healthy subjects (*n* = 19), atherosclerosis patients (*n* = 22), and breast cancer patients (*n* = 18). The obtained results demonstrated that production of proinflammatory TNF*α* was significantly lower in atherosclerosis patients and significantly higher in cancer patients in comparison to healthy subjects. On the other hand, production of anti-inflammatory CCL18 was decreased both in atherosclerosis and in cancer patients [22].

**Figure 3:**
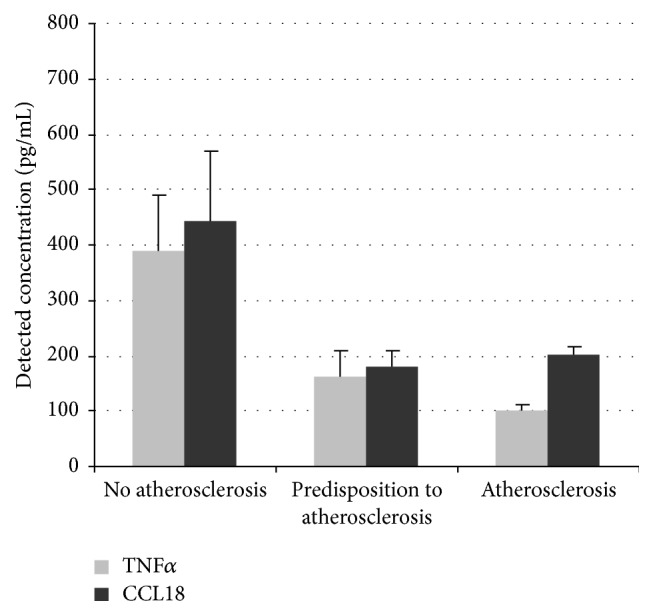
Study of monocyte/macrophage activation in subclinical and clinical atherosclerosis. Monocytes were isolated from the blood of subjects from 3 study groups (*n* = 20 in each group). Cells were stimulated with IFN-*γ* (100 ng/mL) or IL-4 (10 ng/mL). Secretion of TNF*α* and CCL18 was measured by ELISA.

To evaluate the diagnostic potential of macrophages' activation test in asymptomatic atherosclerosis, a study was performed on individuals with predisposition to atherosclerosis (*n* = 21, mean age 63 ± 9 years) and subclinical atherosclerosis (*n* = 21, mean age 62 ± 7 years) in comparison to healthy subjects (*n* = 21, mean age 60 ± 9 years). Predisposition to atherosclerosis and subclinical atherosclerosis were detected by measuring the age-adjusted carotid intima media thickness (cIMT). The analysis of TNF*α* and CCL18 production by stimulated macrophages revealed dramatic individual differences between the analyzed subjects that may reflect the individuals' predisposition to immunopathology. Macrophages from subjects with subclinical atherosclerosis were characterized by especially low degree of activation in response to stimuli [22] (Figure 3). Therefore, the ability of macrophages to polarize towards pro- and anti-inflammatory phenotypes was decreased at early stages of atherosclerosis development, although the causative significance of this observation remains unclear.

## 5. Application of Cellular Tests for Drug Development

Changes of the immune system occur early in many pathological processes, opening the intriguing possibility that patients may benefit from a preventive treatment targeting the underlying immunopathology. Imbalanced macrophage polarization is observed in such conditions as atherosclerosis and cancer. Enhanced monocyte activation may lead to macrophage polarization towards pro- or anti-inflammatory phenotype leading to chronic inflammation and atherosclerosis or to oncopathologies, respectively [22]. Therefore, macrophage depolarization might be exploited for the development of preventive treatment [72]. For instance, it has been demonstrated that depolarization of macrophages from the M2 phenotype was associated with tumor regression [73].

**Figure 4:**
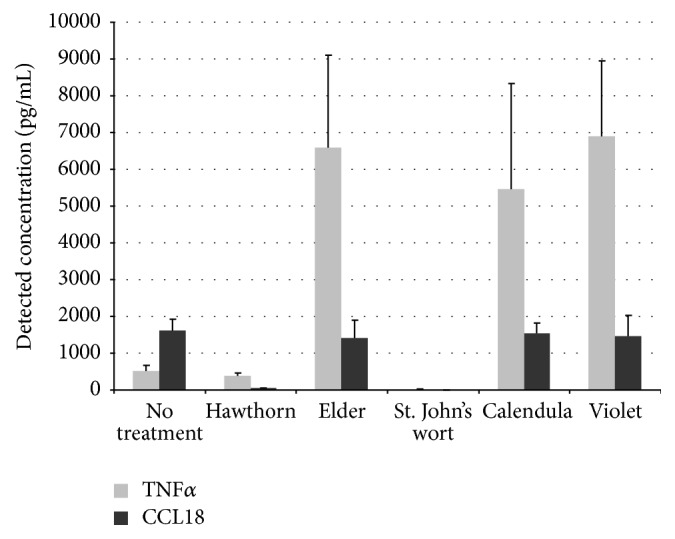
Modulation of macrophage polarization by natural agents. Macrophages were isolated from healthy subjects and brought into primary culture. Macrophages were incubated with extracts of various medicinal plants and stimulated with IFN-*γ* (100 ng/mL) or IL-4 (10 ng/mL). Secretion of TNF*α* and CCL18 was measured by ELISA.

To explore the potential of the macrophage activation test for drug development, the macrophage depolarization effects of herbal extracts were studied on cells obtained from healthy subjects. Plant extracts with immune-modulating properties are widely used in traditional medicine, but their therapeutic potential for modern clinical practice remains to be investigated. The extracts of the following plants with known anti-inflammatory activity were included into the study: flowers of hawthorn (*Crataegus* sp.), elderberry (*Sambucus nigra*), and calendula (*Calendula officinalis*) and herbs of St. John's wort (*Hypericum perforatum*) and violet (*Viola* sp.). Cultured macrophages were exposed to pro- and anti-inflammatory stimuli (IFN-*γ* and IL-4, resp.), and TNF*α* and CCL18 production was measured after 6 days. TNF*α* secretion by IFN-*γ*-stimulated macrophages treated with elderberry, calendula, and violet extracts was 10–13-fold higher than that of untreated stimulated macrophages. On the other hand, hawthorn and St. John's wort extracts significantly inhibited TNF*α* secretion. Extracts of hawthorn and St. John's wort also suppressed the secretion of CCL18 by IL-4-stimulated macrophages (Figure 4). Therefore, St. John's wort and hawthorn extracts appear to be natural agents with immune-modulatory properties that could be used for macrophage depolarization. Importantly, natural agents are characterized by relatively good tolerance and minimal side effects and are therefore especially suitable for long-term therapy, which is necessary for successful immune correction.

One of the therapeutic strategies for treatment of atherosclerosis is the inhibition of intracellular cholesterol accumulation. It is well established that hypercholesterolemia is a potent risk factor for atherosclerosis development [74–76]. However, the accumulating evidence demonstrates that atherogenic potential depends not so much on the total level of cholesterol as on the nature of cholesterol-containing lipoprotein particles that serve as a source of cholesterol storage in the arterial wall. Low-density lipoprotein (LDL) and especially its modifications, such as small dense, oxidized, desialylated, or electronegative LDL, play the key role in atherogenesis, and their levels positively correlate with the disease progression [77–79]. Moreover, modified LDL particles can provoke formation of autoantibodies that initiate the inflammatory response and form highly atherogenic immune complexes with LDL particles [80]. In that regard, LDL composition of blood plasma may be a decisive factor that triggers atherogenesis. The ability of blood serum to induce cholesterol accumulation is referred to as serum atherogenicity. It has been previously demonstrated that serum obtained from atherosclerosis patients caused cholesterol accumulation in cultured cells and therefore was highly atherogenic [81].

**Figure 5:**
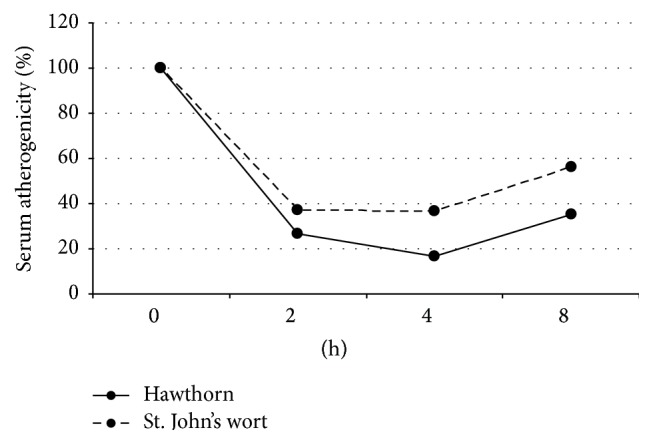
Study of serum atherogenicity in cellular assay. Four patients with atherosclerosis were given water extracts of 8 g of hawthorn berries or 3 g St. John's wort herb. Blood samples were collected before treatment and after 2, 4, and 8 hours and blood serum was added to cultured primary subendothelial intimal cells. Serum atherogenicity was measured as the increase of cholesterol content in cultured cells after 24 h.

Primary culture of human aorta cells can be a useful system for testing various antiatherosclerotic substances. Using this system, atherogenic properties of patient's blood serum can be analyzed before and after drug administration to assess its therapeutic potential [82–84]. Antiatherogenic activity of hawthorn and St. John's wort extracts was tested using the described* ex vivo model*. Serum from study participants was added to cultured subendothelial intimal cells derived from uninvolved human aorta at concentration of 10%, and cholesterol accumulation was measured after 24 h using a previously established method [82]. It was demonstrated that blood serum atherogenicity decreased in study subjects treated with a single dose of hawthorn extract. The observed decrease was 73% and 83% after 2 and 4 hours, respectively, in comparison to the baseline (Figure 5). These results indicate that hawthorn extract may be regarded as a potent antiatherosclerotic agent. On the other hand, St. John's wort extract had no statistically significant effect on cellular cholesterol accumulation.

Taken together, the obtained results demonstrate that the combination of hawthorn and St. John's wort extracts appears promising for the development of antiatherosclerosis therapy. St. John's wort extract was a potent macrophage depolarizing agent, and hawthorn was demonstrated to reduce the serum atherogenicity. Further studies employing macrophage-based cellular test systems will allow identification of novel agents with therapeutic potential.

## 6. Conclusion

Monocyte/macrophage-based test system is a versatile tool to detect immunopathology, including increased monocyte activation and altered polarization of macrophages towards pro- or anti-inflammatory phenotypes. Alterations in monocyte activation and imbalance in macrophage polarization can be associated with a variety of pathological conditions, including different types of cancer and atherosclerosis. Therefore, the development of immunomodulating therapy might contribute significantly to the improvement of the existing treatment strategies. However, the complexity of the immunopathology requires flexible and reliable methods for diagnostics and monitoring of treatment efficiency. The monocyte/macrophage activation test is one of such methods. It was proven to be suitable for the analysis of immunopathology in subclinical atherosclerosis and breast cancer. This method can also be applied for studying numerous other pathologies, where monocyte/macrophage activation is implicated, including other types of cancer, chronic inflammation, and autoimmune disorders. Given the broad spectrum of cytokines that may be analyzed, the described method can be improved to perform a more detailed study of macrophage activation. The application of monocyte/macrophage activation test for drug research was illustrated by screening a series of medicinal plant extracts for antiatherosclerotic activity. Study of macrophage polarization revealed a potent immunomodulatory activity of hawthorn and St. John's wort extracts that might be beneficial for treatment and prevention of atherosclerosis. Together, these results demonstrate the possibilities of macrophage-based cellular tests for diagnostics and drug research in conditions associated with immunopathology.
